# Src-Mediated EGF Receptor Activation Regulates Ozone-Induced Interleukin 8 Expression in Human Bronchial Epithelial Cells

**DOI:** 10.1289/ehp.1307379

**Published:** 2014-10-10

**Authors:** Weidong Wu, Phillip A. Wages, Robert B. Devlin, David Diaz-Sanchez, David B. Peden, James M. Samet

**Affiliations:** 1 School of Public Health, Xinxiang Medical University, Xinxiang, Henan Province, China; 2 Center for Environmental Medicine, Asthma, and Lung Biology, and; 3 Curriculum in Toxicology, University of North Carolina at Chapel Hill, Chapel Hill, North Carolina, USA; 4 Environmental Public Health Division, National Health and Environmental Effects Research Laboratory, U.S. Environmental Protection Agency, Chapel Hill, North Carolina, USA

## Abstract

**Background:**

Human exposure to ozone (O_3_) results in pulmonary function decrements and airway inflammation. The mechanisms underlying these adverse effects remain unclear. Epidermal growth factor receptor (EGFR) plays an important role in the pathogenesis of lung inflammation.

**Objective:**

We examined the role of EGFR activation in O_3_-induced expression of the chemokine interleukin 8 (IL-8) in human bronchial epithelial cells (HBEC).

**Methods:**

We detected phosphorylated EGFR using immunoblotting. EGFR dimerization was examined through cross-linking reaction and immunoblotting, and levels of IL-8 protein were measured using ELISA.

**Results:**

Exposure to O_3_ (0.25–1.0 ppm) induced rapid and marked increase in EGFR phosphorylation at the autophosphorylation site Y1068 and the transphosphorylation site Y845, implicating the involvement of Src kinase. Further investigation showed that O_3_ stimulation induced phosphorylation of Src at Y416, indicative of Src activation. Pharmacological inhibition of Src kinase activity abrogated O_3_-induced EGFR phosphorylation at tyrosines 1068 and 845. Moreover, pretreatment of BEAS-2B cells with inhibitor of either EGFR or Src kinase activities significantly blocked O_3_-induced IL-8 expression.

**Conclusion:**

Conclusion: O_3_ exposure increased IL-8 expression through Src-mediated EGFR transactivation in HBEC.

**Citation>:**

Wu W, Wages PA, Devlin RB, Diaz-Sanchez D, Peden DB, Samet JM. 2015. Src-mediated EGF receptor activation regulates ozone-induced interleukin 8 expression in human bronchial epithelial cells. Environ Health Perspect 123:231–236; http://dx.doi.org/10.1289/ehp.1307379

## Introduction

Ozone (O_3_) is formed by the photochemical reaction of sunlight with nitrogen oxides, facilitated by the presence of a variety of volatile organic compounds. Natural background concentrations of ground-level ozone are typically around 30–100 μg/m^3^. However, short-term (1-hr) mean ambient concentrations in urban areas may exceed 300–800 μg/m^3^ (World Health Organization 1979). Both natural and anthropogenic sources contribute to the emission of ground-level O_3_ precursors, and the composition of emissions may show large variations across locations (World Bank Group 1998). Motor vehicles are the main anthropogenic sources of ground-level O_3_ precursors (Devlin et al. 1994; Seltzer et al. 1986).

A large volume of information on the health impacts of ground-level ozone is derived from animal studies, whereas a more limited number of investigations have concentrated on population and controlled human studies focused on short-term exposures. Clinical studies on asthmatic and nonasthmatic adults have demonstrated that acute exposure to O_3_ results in decreases in lung function, enhanced allergen-induced bronchoconstriction, and increases in airway inflammation typified by an influx of neutrophils (Alexis et al. 2004, 2008, 2010; Hernandez et al. 2010; Holz et al. 1999; Jörres et al. 2000). Similarly, short-term exposure to elevated levels of O_3_ leads to an early inflammatory response characterized by neutrophil accumulation in several animal models (Driscoll et al. 1993; Kleeberger and Hudak 1992; Seltzer et al. 1986; Zhao et al. 1998).

Interleukin 8 (IL-8) is a potent neutrophil activator and chemotaxin that is often used as a biological marker of environmentally induced pulmonary inflammation (Kunkel et al. 1991; Standiford et al. 1993). O_3_ inhalation induces airway epithelial damage and increased release of proinflammatory mediators, including IL-8, in human bronchoalveolar lavage fluid (Bosson et al. 2003; Krishna et al. 1998). *In vitro* exposure to O_3_ has been shown to induce IL-8 production in human bronchial epithelial cells (HBEC) (Bayram et al. 2001; Devlin et al. 1994; Rusznak et al. 1996). However, the mechanisms that regulate O_3_-induced IL-8 expression have not been fully elucidated.

The expression of the *IL-8* gene in HBEC is known to be regulated through both message stabilization and transcriptional activation that is modulated by signaling pathways that include growth factor receptors (Khabar 2010; Standiford et al. 1993). The activation of the epidermal growth factor receptor (EGFR) is a pivotal event in normal and pathophysiological conditions leading to the initiation of multiple signaling pathways that lead to alterations in gene expression (Takeyama et al. 2001). EGFR is a single transmembrane protein that possesses intrinsic tyrosine kinase activity, which can be directly activated or transactivated in response to a variety of stimuli (Gschwind et al. 2001). The cytoplasmic region of human EGFR contains an intrinsic tyrosine kinase (697–955) followed by a 231-residue-long COOH-terminal tail, which contains multiple tyrosine residues that function as phosphorylation sites (Xia et al. 2002). Five sites of *in vivo* autophosphorylation have been identified in the EGFR: three major sites (tyrosines 1068, 1148, and 1173) and two minor sites (tyrosines 992 and 1086) (Downward et al. 1984; Margolis et al. 1989). The binding of phosphorylated EGFR tyrosines with downstream signaling proteins initiates the simultaneous activation of multiple signaling cascades that culminate in a broad range of cellular responses spanning proliferation, migration, protein secretion, differentiation, and oncogenesis (Wells 1999).

Previous studies have demonstrated that epithelial expression of the EGFR and its ligands, including EGF and transforming growth factor α, were all significantly increased in nasal biopsy specimens collected following O_3_ exposure of human volunteers, suggesting a positive correlation between EGFR expression and the increase in neutrophil numbers in the nasal epithelium (Polosa et al. 2004). Another study using human epidermal keratinocytes showed that exposure to O_3_ resulted in increased phosphorylation of EGFR (Afaq et al. 2009). In the present study, we investigated the effect of O_3_ stimulation on EGFR phosphorylation and its relationship with IL-8 expression in HBEC. We report here that the cytosolic tyrosine kinase Src can regulate EGFR activity, further modulating O_3_-induced IL-8 expression.

## Materials and Methods

*Reagents*. Bis[sulfosuccinimidyl]suberate (BS3), PP2, and bosutinib (the latter two being Src kinase inhibitors) and compound 56 (C56; an EGFR inhibitor) were obtained from Calbiochem (San Diego, CA, USA). The rabbit antibodies against phospho (p)-EGFR (Y1068), p-EGFR (Y845), p-Src(Y416), pan-EGFR, and pan-Src were purchased from Cell Signaling Technology (Beverly, MA, USA). Horseradish peroxidase (HRP)-conjugated goat anti-rabbit antibody was obtained from Santa Cruz Biotechnology (Santa Cruz, CA, USA). The lactate dehydrogenase (LDH) cytotoxicity detection kit was obtained from TAKARA Bio Inc. (Mountain View, CA, USA), and the IL-8 ELISA assay kit was purchased from eBioscience (San Diego, CA, USA).

*Cell culture and O_3_ exposure*. The BEAS-2B cell line was derived by transforming HBEC with an adenovirus 12-simian virus 40 construct (Reddel et al. 1988). BEAS-2B cells (passages 70–80) were cultured in supplemented keratinocyte basal medium as described previously (Wu et al. 2011). The cells were placed in 6-well culture plates (Costar, Cambridge, MA, USA) and grown to confluence.

Normal human bronchial epithelial (NHBE) cells were obtained from normal adult human volunteers by brush biopsy of the mainstem bronchus using a cytology brush during fiberoptic bronchoscopy; the procedure was conducted under a protocol approved by the Committee on the Protection of the Rights of Human Subjects at the University of North Carolina at Chapel Hill. Human participants gave written informed consent prior to the study. NHBE cells were initially plated in supplemented bronchial epithelial cell basal medium (Tal et al. 2006). Confluent cells were split and placed on the Transwell® permeable supports (Corning, Tewksbury, MA, USA) for air–liquid interface (ALI) culture before O_3_ exposure.

A431 cells were obtained from the Lineberger Cancer Center Tissue Culture Facility at the University of North Carolina at Chapel Hill. A431 cells were cultured on plastic flasks in Dulbecco’s minimum essential medium with high glucose supplemented with 10% fetal bovine serum and gentamicin (5 μg/mL).

Prior to exposure of BEAS-2B or A431 cells, 0.5 mL of media was placed in each well of the 6-well plates. In some experiments, BEAS-2B cells were pretreated with 1 μM C56, 10 μM PP2, or bosutinib (5 or 10 μM) for 30 min before O_3_ exposure. After exposure, the cells were lysed and the cell lysates subjected to immunoblotting for measurement of p-EGFR (Y1068), or p-EGFR (Y845). The supernatants of cell medium were collected for measurement of IL-8 protein. Exposure to O_3_ or clean air was conducted using a rotating inclined platform in specially designed *in vitro* exposure chambers operated by the U.S. Environmental Protection Agency Environmental Public Health Division as described previously (Devlin et al. 1994). The exposure atmosphere contained 5% CO_2_; the relative humidity was maintained at > 95%; and the temperature was 37°C throughout the exposure. Conditions in the air control chamber were identical except for the absence of O_3_.

In the case of the ALI-cultured NHBE, the cells were exposed without media on the apical surface without rotation of the platform. Air controls were run simultaneously in an identical duplicate chamber in which no ozone was introduced.

*Analysis of EGFR dimerization*. The EGFR dimerization assay was performed according to a previously described method (Samet et al. 2003). Briefly, subconfluent cells deprived of serum for 12–18 hr were exposed to 1 ppm O_3_ or air alone (control) for 30 min in an O_3_ exposure chamber as described above. After this exposure, cells were incubated with 10 ng/mL EGF for 5 min. The cells were then treated with 2.5 mM crosslinker BS3 in phosphate-buffered saline (PBS) for 30 min at room temperature. The cross-linking reaction was stopped by incubating cells with PBS containing 20 mM Tris, pH 7.5, for 15 min. The cells were then scraped into 1 mL of PBS, and protein extracts were prepared and subjected to immunoblotting using a mouse anti-human EGFR antibody cocktail (Neomarkers, Fremont, CA, USA) that recognizes the extracellular domain of the EGFR.

*Immunoblotting*. The cells exposed to 0.25–1.0 ppm O_3_ for 0–120 min were washed twice with ice-cold PBS and then lysed in RIPA buffer as described previously (Wu et al. 2011). The supernatants of cell lysates were subjected to SDS-PAGE analysis. Proteins were transferred onto nitrocellulose membrane, which was then blocked with 5% nonfat milk, washed briefly, incubated with primary antibody at 4°C overnight, and then incubated with the corresponding HRP-conjugated secondary antibody for 1 hr at room temperature. Immunoblot images were detected using chemiluminescence reagents and a Fujifilm LAS-3000 imaging system (Fuji Medical Systems USA, Stamford, CT, USA), and the images were digitized for quantification using MultiGauge v3.1 software (Fuji Medical Systems USA). The optical density (OD) of the phosphor-specific band was expressed as a fraction (% P) of the total OD (i.e., phospho + non-phospho bands) for the species of interest.

*ELISA (enzyme linked immunosorbent assay).* After exposure to 1 ppm O_3_ for 2 hr, cell culture media were collected and centrifuged. Levels of IL-8 protein in the supernatants were measured using an IL-8 ELISA assay kit following the manufacturer’s instructions (eBiosciences, San Diego, CA, USA).

*Statistical analysis*. Data are presented as mean ± SE. Data comparisons were carried out using one-way analysis of variance (ANOVA) followed by Dunnett’s post-test and two-tailed Student’s *t*-test, with the overall α level set at 0.05.

## Results

**Figure 1:**
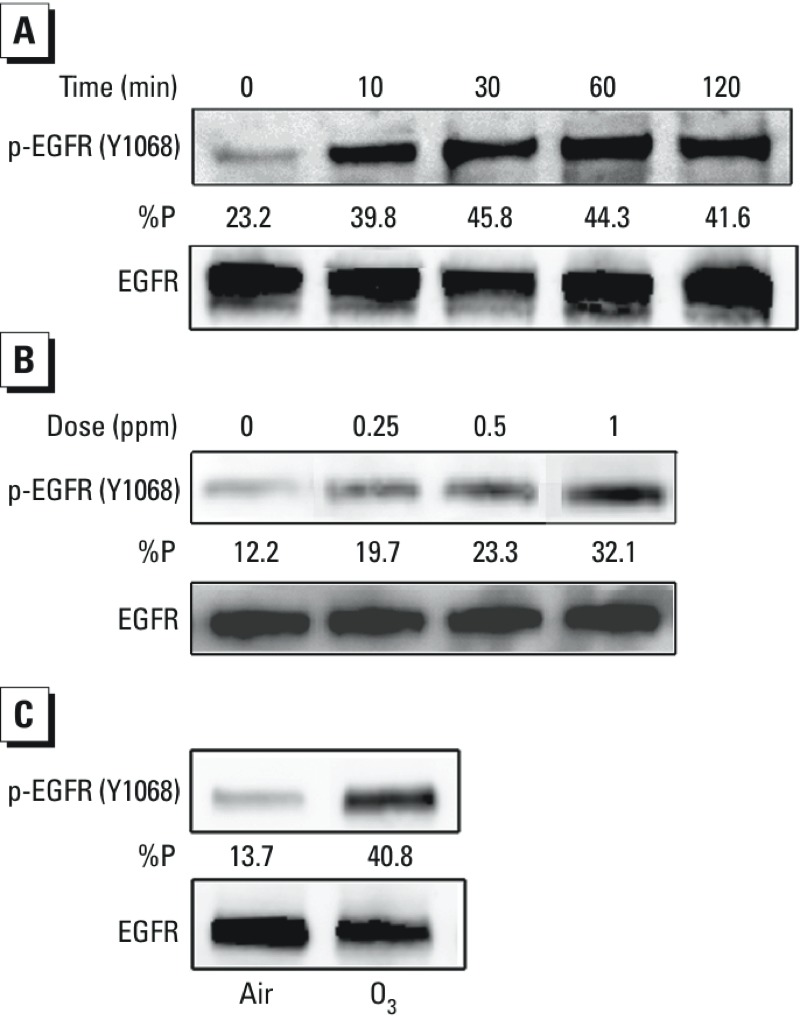
Effect of O_3_ exposure on phosphorylation of EGFR (Y1068) in HBEC. (*A*) BEAS-2B cells grown to confluence were exposed to 1 ppm O_3_ for 0–120 min. (*B*) BEAS-2B cells grown to confluence were exposed to various concentrations of O_3_ for 2 hr. (*C*) NHBE cells were cultured in an ALI system and exposed to 1 ppm O_3_ for 30 min. In each case (*A–C*), protein was extracted from the cells and subjected to SDS-PAGE followed by immunoblotting using a phospho-specific anti-EGFR antibody and then a pan-EGFR antibody. %P indicates the optical density of the p-EGFR band as a fraction of the total EGFR signal (p-EGFR + EGFR). Data shown are representative of three separate experiments.

*O_3_ exposure and induction of phosphorylation of EGFR (Y1068) in HBEC*. Preliminary experiments determined O_3_ concentrations and exposure times that did not result in significant cytotoxicity, as determined by LDH release (data not shown). Phosphorylation of EGFR is an indicator of its activation. O_3_-induced EGFR phosphorylation has been shown previously in human skin cells (Afaq et al. 2009). Here, we examined whether O_3_ exposure could affect EGFR phosphorylation in HBEC. As shown in Figure 1A,B, O_3_ exposure induced a concentration- and time-dependent increase in EGFR phosphorylation at tyrosine 1068 (Y1068) in BEAS-2B cells. Exposure to O_3_ was also observed to increase EGFR (Y1068) phosphorylation in ALI-cultured primary HBEC, demonstrating that this effect of O_3_ is not limited to transformed cells (Figure 1C).

**Figure 2:**
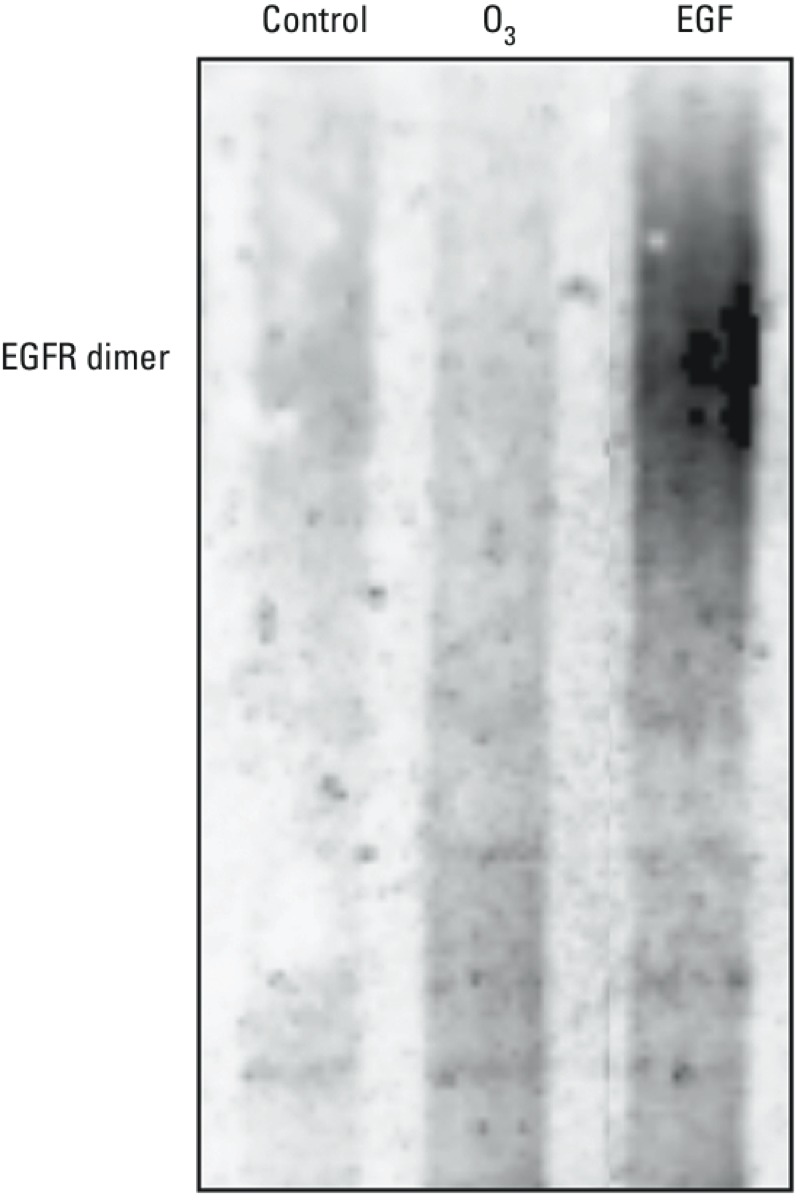
O_3_ exposure and EGFR dimerization in A431 cells. Confluent A431 cells were exposed to air (control) or 1 ppm O_3_ for 30 min, or to 10 ng/mL EGF for 5 min. The cells were then exposed to saline or the cross-linking agent BS3, and total protein extracts were extracted and subjected to Western blotting using anti-EGFR antibody.

*O_3_ exposure and induction of EGFR dimerization*. The finding that O_3_ exposure induces the phosphorylation of EGFR at the putative autophorylation site Y1068 (Downward et al. 1984) suggested the possibility that O_3_ activates the receptor through a mechanism that mimics ligand binding of the extracellular domain on the EGFR. To investigate this potential mechanism of activation of O_3_-induced EGFR, we measured EGFR dimerization, a marker of ligand-dependent activation of the EGFR, in the EGFR overexpressing cell line A431. As shown in Figure 2, relative to the air-treated control, treatment of A431 cells with the natural ligand EGF induced dimerization of EGFR within 5 min. In marked contrast, EGFR dimerization was not detectable in A431 cells exposed to 1 ppm O_3_ for 30 min, indicating that O_3_ exposure did not cause EGFR activation through a ligand-like mechanism.

**Figure 3:**
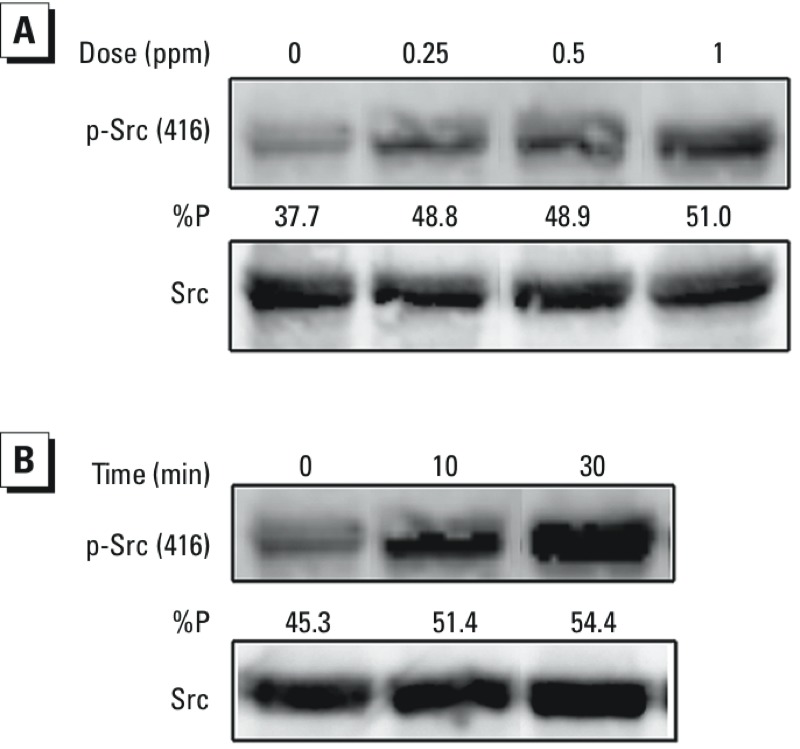
O_3_ exposure and phosphorylation of Src (Y416) in BEAS-2B cells. BEAS-2B cells grown to confluence were exposed to various concentrations of O_3_ for 2 hr (*A*) or to 1 ppm O_3_ for 0–30 min (*B*). In each case, protein was extracted from the cells and subjected to SDS-PAGE followed by immunoblotting using a phospho-specific anti-Src (Y416) antibody and then a pan-Src antibody. %P indicates the optical density of the p-Src band as a fraction of the total Src signal (p-Src + Src). Data shown are representative of three separate experiments.

*O_3_ exposure and induction of Src-​dependent phosphorylation of the EGFR in HBEC*. We next examined the potential of EGFR activation through transphosphorylation by a kinase intermediate. We have shown previously that cytosolic tyrosine kinase Src is involved in EGFR transactivation in HBEC exposed to another oxidant stressor (Wu et al. 2002). Therefore, we examined the role of Src kinase in O_3_-induced EGFR phosphorylation by first determining the effect of O_3_ exposure on phosphorylation of Src at tyrosine 416, a specific activation site in the SH1 domain of c-Src (Dorsam et al. 2005), in O_3_-exposed BEAS-2B cells. Exposure to 0.25–1.0 ppm O_3_ for 120 min induced a concentration-dependent increase in the phosphorylation of Src (Y416) (Figure 3A). In addition, a time-dependent increase in Src (Y416) phosphorylation was observed in response to exposure to 1 ppm O_3_ (Figure 3B).

**Figure 4:**
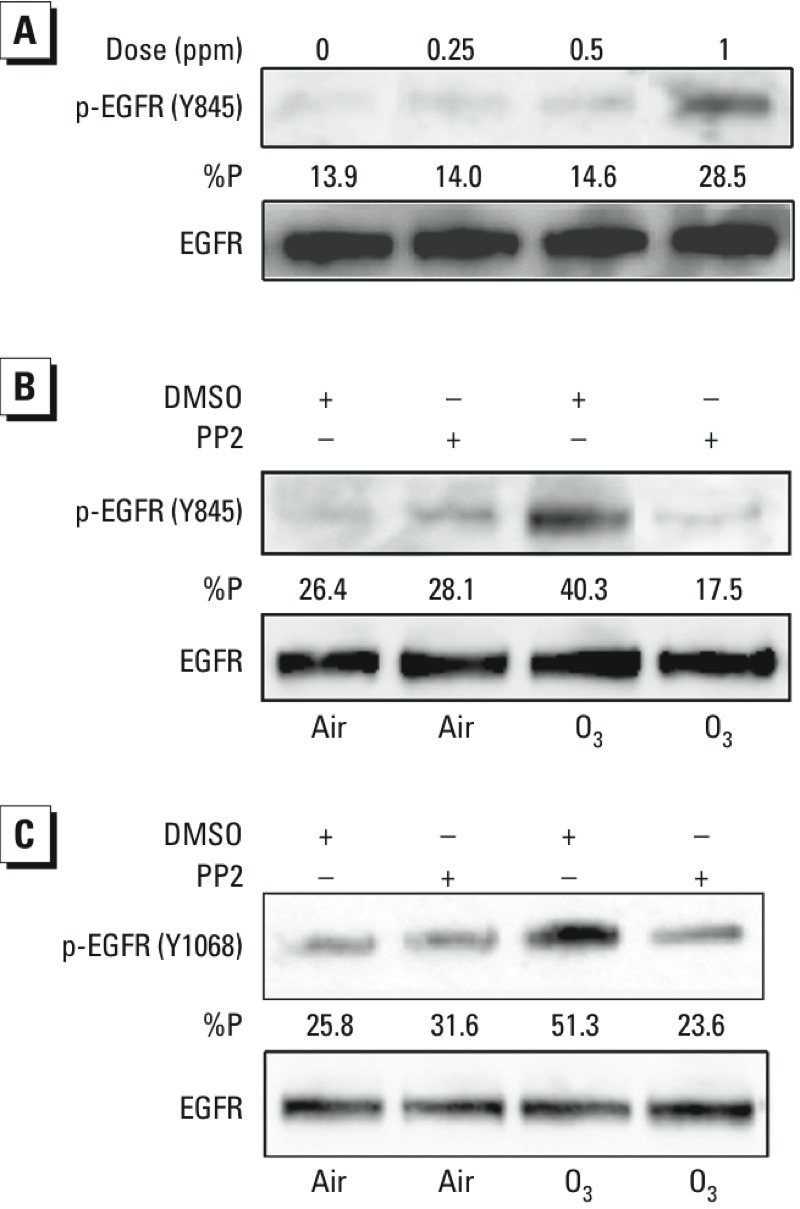
Src kinase and O_3_-induced phosphorylation of EGFR in HBEC. (*A*) BEAS-2B cells grown to confluence were exposed to various concentrations of O_3_ for 2 hr. BEAS-2B cells (*B*) or ALI-cultured NHBE cells (*C*) were pretreated with vehicle (0.1% DMSO) or the Src kinase inhibitor PP2 (10 μM) for 30 min prior to exposure to 1 ppm O_3_ for 1 hr. In each case (*A–C*), protein was extracted from the cells and subjected to SDS-PAGE followed by immunoblotting using phospho-specific EGFR antibodies and then a pan-EGFR antibody. %P indicates the optical density of the p-EGFR band as a fraction of the total EGFR signal (p-EGFR + EGFR). Data shown are representative of three separate experiments.

Tyrosine 845 on the EGFR has been reported to be a site of transphosphorylation by Src kinase (Tice et al. 1999). As shown in Figure 4A, exposure to 0.25–1.0 ppm O_3_ for 120 min induced phosphorylation of EGFR (Y845), an effect that was ablated by pretreatment of the cells with the Src kinase inhibitor PP2 (Figure 4B). Similarly, inhibition of Src kinase activity also blunted O_3_-induced phosphorylation of EGFR at Y1068 in NHBE cells (Figure 4C). Pretreatment of BEAS-2B cells with the structurally unrelated Src kinase inhibitor bosutinib corroborated these findings (see Supplemental Material, Figure S1). We previously observed that exposure to Zn^2+^ induced Src-dependent phosphorylation of EGFR (Wu et al. 2002). As we expected in the present study, Src inhibition with bosutinib effectively reduced Zn^2+^-induced phosphorylation of EGFR in BEAS-2B cells (see Supplemental Material, Figure S2). Taken together, these data implicated Src kinase in O_3_-induced EGFR transactivation at both transphosphorylation and autophosphorylation sites in HBEC.

**Figure 5:**
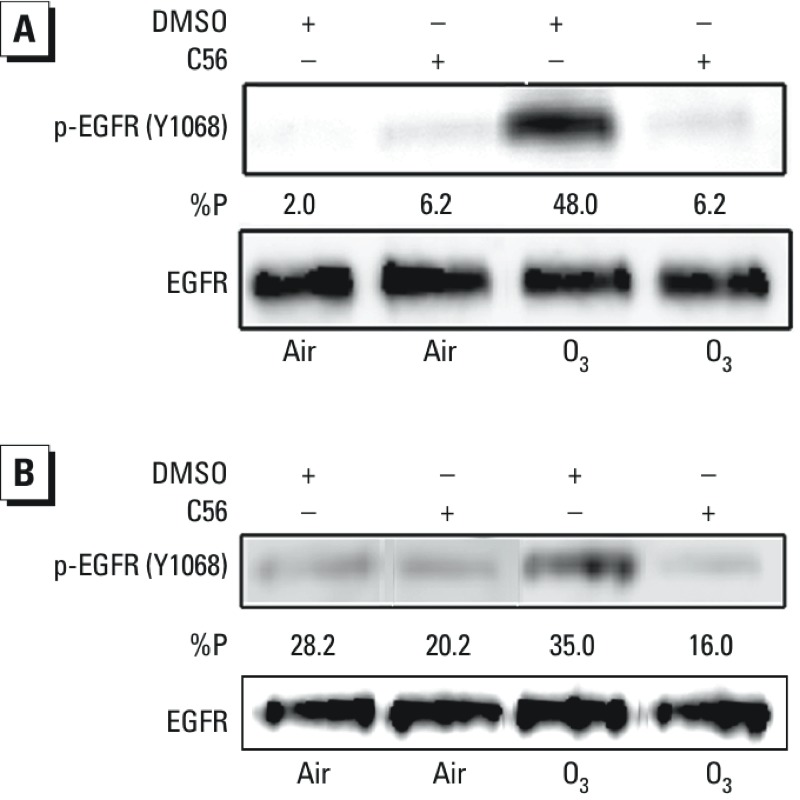
Abrogation of O_3_-induced phosphorylation of EGFR (Y1068) is by the EGFR inhibitor C56. BEAS-2B cells (*A*) or ALI-cultured NHBE cells (*B*) were pretreated with vehicle (0.1% DMSO) or C56 (1 μM) for 30 min before exposure to 1 ppm O_3_ for 1 hr. Cells were lysed, and phosphorylation of EGFR (Y1068) was determined as described in “Materials and Methods.” Data shown are representative of three separate experiments.

*EGFR kinase–dependent EGFR (Y1068) phosphorylation in O_3_-exposed HBEC*. Previous studies have shown that phosphorylation of EGFR (Y1068) can occur in the presence or absence of EGFR kinase activity (Mueller et al. 2008; Wu et al. 2005). To determine whether Src-mediated EGFR (Y1068) phosphorylation is EGFR kinase-dependent, we pretreated BEAS-2B cells (Figure 5A) or ALI-cultured primary HBEC (Figure 5B) with vehicle (0.1% DMSO) or the specific EGFR kinase activity inhibitor C56 (1 μM) for 30 min before exposure to 1 ppm O_3_ for 1 hr. As shown in Figure 5, inhibition of EGFR kinase activity in HBEC abrogated O_3_-induced EGFR (Y1068) phosphorylation, implying that O_3_-induced phosphorylation of EGFR at Y1068 requires the intrinsic kinase activity of the EGFR.

**Figure 6:**
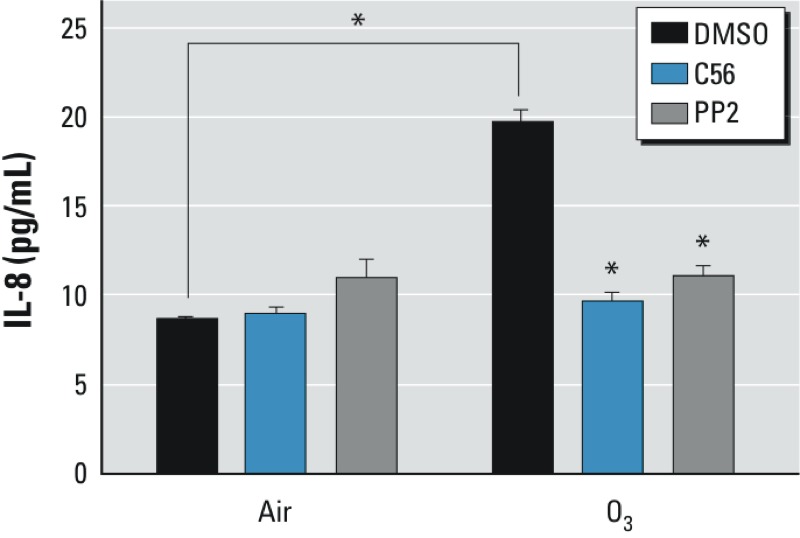
Effects of C56 and PP2 on O3-induced IL-8 expression in BESA-2B cells. BEAS-2B cells were pretreated with vehicle (0.1% DMSO), PP2 (10 μM), or C56 (1 μM) for 30 min before exposure to 1 ppm O_3_ for 2 hr. Culture media were collected, and IL-8 protein was examined in the supernatants using ELISA. *p < 0.05 (*n* = 3) compared with matched air controls, by Student’s *t*-test.

*Requirement of EGFR and Src kinase activities for O_3_-induced IL-8 expression in HBEC*. Previous studies have shown that exposure to O_3_ results in increased expression of proinflammatory mediators, including IL-8 in HBEC (Bayram et al. 2001; Devlin et al. 1994; Hiltermann et al. 1998; McKinnon et al. 1992). To determine the roles of EGFR and Src activation in proinflammatory response of HBEC to O_3_ exposure, we examined the effect of EGFR or Src inhibition on O_3_-induced IL-8 expression in BEAS-2B cells. As shown in Figure 6, pretreatment of BEAS-2B cells with either an EGFR or Src kinase activity inhibitor was able to abrogate IL-8 expression induced by exposure to 1 ppm O_3_ for 2 hr.

## Discussion

The mechanisms for the transactivation of EGFR vary with the cell type and stimulus. Previous studies have shown that EGFR is involved in signaling networks activated by a number of stimuli that do not interact directly with this receptor (Carpenter 1999). These stimuli include G protein–coupled receptor agonists (Daub et al. 1997), calcium (Dethlefsen et al. 1998), and ultraviolet irradiation (Dent et al. 1999). The results of the present study show that O_3_ exposure transactivates EGFR through a mechanism that depends on the activation of the cytosolic tyrosine kinase Src, leading to elevated expression of IL-8.

The dependency of O_3_-induced EGFR activation on Src activation in HBEC is supported by the findings of O_3_-induced phosphorylation of Src at Y416, the phosphorylation of EGFR at the transactivation site Y845, and the inhibitory effect of Src kinase inhibitors on EGFR phosphorylation. The mechanisms involved in O_3_-induced Src activation are currently unknown. In a previous study, we observed that O_3_ exposure induced reactive oxygen species (ROS) production in BEAS-2B cells (Wu et al. 2011). Thus, O_3_-induced excessive production of ROS and nitrogen intermediates (Hulo et al. 2011) might be able to modify tyrosine residues, altering phosphorylation of many protein kinases involved in cell signalling (Akhand et al. 1999). Another mechanism underlying O_3_-induced Src activation may involve loss of homeostatic phosphatase activity. We previously observed that loss of protein tyrosine phosphatase (PTP) activity was responsible for the initiation of EGFR signaling in HBEC exposed to zinc or diesel exhaust particles (Tal et al. 2006, 2008). PTPs are redox-sensitive proteins; their active-site cysteines are the targets of specific oxidation by various oxidants (Giannoni et al. 2005). In this regard, our observation that another electrophilic insult, Zn^2+^, can induce Src-dependent EGFR transphosphorylation (Wu et al. 2002) is consistent with our previous report of Zn^2+^-induced inhibition of PTPs (Tal et al. 2006), and may reflect a uniform mechanism to oxidant stress that is also relevant to other environmental electrophiles. Additional studies will be required to investigate the role of PTP activity in O_3_-induced activation of Src leading to EGFR activation and IL-8 expression.

EGFR (Y845) is particularly interesting because of its location within the activation loop of the tyrosine kinase domain of the EGFR. This tyrosine residue is highly homologous to tyrosines that are sites of autophosphorylation found in the kinase domains of other tyrosine kinases that have been shown to be critical to their activation (Biscardi et al. 2000). Unlike the tyrosines in these other kinases, EGFR (Y845) is not an autophosphorylation site and does not need to be phosphorylated for the kinase to be active (Tice et al. 1999). However, phosphorylation of EGFR (Y845) has been proposed as a direct substrate of Src (Tice et al. 1999). Our previous study (Wu et al. 2002) demonstrated that mutating this tyrosine to a nonphosphorylatable phenylalanine led to an abrogation of zinc-induced Ras activation in fibroblasts. In a separate study we observed that the Src kinase inhibitor PP2 significantly blocked zinc-induced phosphorylation of EGFR (Y845) in A431 cells (Samet et al. 2003). Similarly, in the present study PP2 or bosutinib pretreatment markedly inhibited O_3_-induced EGFR (Y845) phosphorylation. Taken together, these data suggest that EGFR (Y845) phosphorylation is critical to EGFR/c-Src synergy and cross talk.

Nevertheless, there is also evidence to suggest that EGFR (Y845) is not the only target site for Src kinase. For example, a study in A431 cells showed that zinc ions can induce phosphorylation of EGFR (Y1068) and EGFR (Y845) that is Src dependent but EGFR kinase independent (Samet et al. 2003). Amos et al. (2005) demonstrated that Src kinase is required for phorbol 12-myristate 13-acetate–induced EGFR (Y1068) phosphorylation using PP2 and siRNA against c-Src. These studies suggest that Src kinase can directly phosphorylate EGFR (Y1068). In contrast, the present study indicates that Src-regulated EGFR (Y1068) phosphorylation is mediated by EGFR kinase because the EGFR kinase inhibitor C56 significantly blocked O_3_-induced EGFR (Y1068) phosphorylation in BEAS-2B cells. Thus, we infer that O_3_ stimulation first activates Src, which in turn causes EGFR (Y845) phosphorylation. Phosphorylated EGFR (Y845) located in the EGFR kinase domain facilitates the modification of EGFR kinase domain conformation, leading to its activation and the subsequent autophosphorylation of EGFR (Y1068). This hypothesis could be examined using cells expressing kinase-inactive or Y845-mutated EGFR. In addition, oxidative stress has been reported to induce ligand-independent EGFR activation through a conformational modification of the intracellular kinase domain under conditions in which the c-Src is physically bound to EGFR (Filosto et al. 2011). In addition, the extreme reactivity of O_3_ may have led to an underestimation of cytotoxicity in the present study, leaving open the possibility that the initiation of signaling is secondary to the induction of cellular injury. Additional studies will be required to test these possible mechanisms of EGFR activation in O_3_-exposed HBEC.

EGFR has been shown to be involved in signaling leading to increased IL-8 production by human airway epithelial cells in chronic inflammatory airway diseases (Hamilton et al. 2003; Richter et al. 2002). In the present study we observed that Src-regulated EGFR activation was necessary for O_3_-induced IL-8 expression in HBEC. Additional mechanisms involved in O_3_-induced EGFR activation leading to IL-8 remain to be elucidated. The traditional cytoplasmic EGFR-initiated route involves transduction of mitogenic signals through activation of several signaling cascades, such as phospholipase Cγ, protein kinase C, mitogen-activated protein kinases, phosphatidylinositol-3-kinase, and signal transducer and activator of transcription (STATs) (Bae et al. 1997; Cattaneo et al. 2011; Lo and Hung 2006; Martínez-Carpio and Trelles 2010). In the nuclear pathway, activated EGFR undergoes fast nuclear translocation, where it physically or functionally interacts with other transcription factors possessing DNA-binding activity and STAT3, leading to up-regulation of genes involved in a host of cellular responses that include IL-8 expression (Lin et al. 2001; Lo and Hung 2006; Xu et al. 2006).

## Conclusion

Our findings indicate that exposure to O_3_ resulted in Src-dependent EGFR activation leading to a proinflammatory response that included IL-8 expression in human airway epithelial cells. Given that O_3_ exposure is a widespread public health concern, characterization of the mechanisms underlying O_3_-induced airway inflammation will yield insights useful in the design of preventive and therapeutic interventions.

## Supplemental Material

(360 KB) PDF
