# Supplementary material for: Src-Mediated EGF Receptor Activation Regulates Ozone-Induced Interleukin 8 Expression in Human Bronchial Epithelial Cells
Source: Environ Health Perspect. 2014 Oct 10;123(3):231–6. doi: 10.1289/ehp.1307379 (PMC4348738; doi:10.1289/ehp.1307379)
Supplement: (360 KB) PDF [file ehp.1307379.s001.508.pdf]

Environmental Health Perspectives doi:10.1289/ehp.1307379

Supplemental Material

Src-Mediated EGF Receptor Activation Regulates Ozone-Induced Interleukin 8 Expression in Human Bronchial Epithelial Cells

Weidong Wu, Phillip A. Wages, Robert B. Devlin, David Diaz-Sanchez, David B. Peden, and James M. Samet

Figure S1

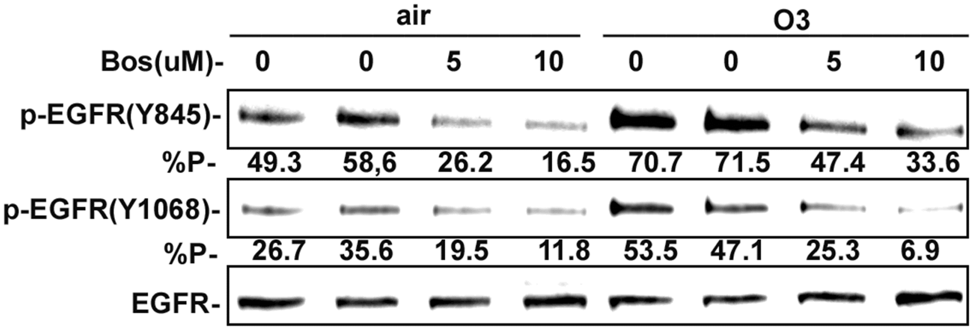

Figure S1. Src kinase is required for O_3-induced EGFR phosphorylation. BEAS-2B cells grown to confluence were pretreated with vehicle control (0.1% DMSO), or the Src kinase inhibitor Bosutinib (5 or 10 μM) for 30 min prior to exposure to 1 ppm O_3 for 1h. Proteins were extracted from the cells and subjected to SDS-PAGE followed by immunoblotting using phospho-specific EGFR antibodies, followed by a pan-EGFR antibody. % P indicates the optical density of the p-EGFR band as a fraction of the total EGFR signal (p-EGFR+EGFR). Data shown are representative of three separate experiments. *, indicates $P<0.05$ compared to O_3 in DMSO group.

Figure S2

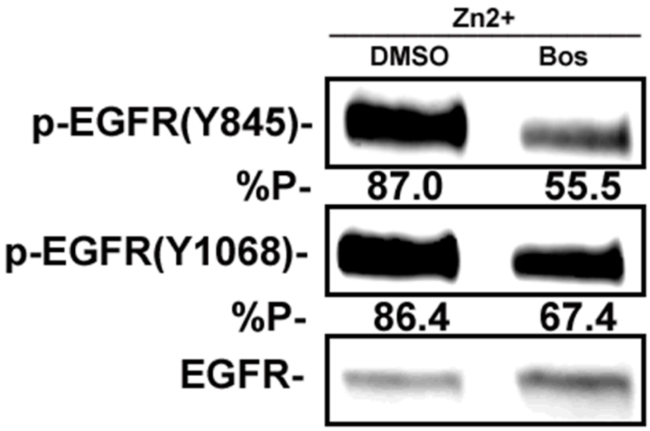

Figure S2. Src kinase is required for Zn2+-induced phosphorylation of EGFR in BEAS-2B cells. BEAS-2B cells grown to confluence were pretreated with vehicle control (0.1% DMSO), or the Src kinase inhibitor Bosutinib (10 µM) for 30 min prior to exposure to exposed to 100 µM $ZnSO_4$ for 1 h. Extracted cellular proteins were subjected to SDS-PAGE followed by immunoblotting using phospho-specific EGFR antibodies, followed by a pan--EGFR antibody. % P indicates the optical density of the p-EGFR band as a fraction of the total EGFR signal (p-EGFR+EGFR). Data shown are representative of three separate experiments.
